# Patient and provider perspectives on adherence to and care coordination of lynch syndrome surveillance recommendations: findings from qualitative interviews

**DOI:** 10.1186/s13053-018-0090-4

**Published:** 2018-05-10

**Authors:** Jennifer L. Schneider, Katrina A. B. Goddard, Kristin R. Muessig, James V. Davis, Alan F. Rope, Jessica E. Hunter, Susan K. Peterson, Louise S. Acheson, Sapna Syngal, Georgia L. Wiesner, Jacob A. Reiss

**Affiliations:** 1 Center for Health Research, Kaiser Permanente Northwest, Portland, OR USA; 2 Department of Behavioral Science, University of Texas MD Anderson Cancer Center, Houston, TX USA; 3 Case Western Reserve University, University Hospitals Cleveland Medical Center, Cleveland, OH USA; 4 Dana-Farber Cancer Institute, Boston, MA USA; 5 Vanderbilt Hereditary Cancer Program, Department of Medicine, Vanderbilt-Ingram Cancer Center, Vanderbilt University, Nashville, TN USA

**Keywords:** Lynch syndrome, Surveillance recommendations, Patient and provider perspectives, Qualitative

## Abstract

**Background:**

Patients with a genetic variant associated with Lynch syndrome (LS) are recommended to undergo frequent and repeated cancer surveillance activities to minimize cancer-related morbidity and mortality. Little is known about how patients and primary care providers (PCPs) track and manage these recommendations. We conducted a small exploratory study of patient and PCP experiences with recommended LS surveillance activities and communication with family members in an integrated health care system.

**Methods:**

We used in-depth interviews with patients and providers to understand how surveillance is coordinated and monitored following confirmation of LS. We recruited patients with a range of ages/gender, and providers with at least at least one patient with a molecular diagnosis of LS. All interviews were recorded, transcribed, and content analyzed by a trained qualitative methodologist.

**Results:**

Twenty-two interviews were completed with 12 patients and 10 providers. Most patients (10) had detailed knowledge of surveillance recommendations, but were less sure of time intervals. While all patients reported receiving initial education about their surveillance recommendations from a genetic counselor, seven did not follow-up with a genetic counselor in subsequent years. A third of patients described taking sole responsibility for managing their LS surveillance care. Lack of routine communication from the health system (e.g., prompts for surveillance activities), and provider engagement were surveillance barriers. PCPs were generally aware of LS, but had limited familiarity with surveillance recommendations. Most PCPs (7) viewed LS as rare and relied on patient and specialist expertise and support. Providers typically had 1 patient with LS in a panel of 1800 patients overall. Providers felt strongly that management of LS should be coordinated by a dedicated team of specialists. Most patients (92%) had at least one family member that sought LS testing, and common barriers for family members included lack of insurance, affordability, and fear of result.

**Conclusion:**

The maximal benefits of screening for confirmation of LS will only be realized with adherence to recommended preventive care. Important factors to ensure patients receive recommended LS care include a comprehensive and coordinated monitoring program that includes reminder prompts, and increased PCP education of LS and associated surveillance recommendations.

## Background

Lynch syndrome (LS) is the most common form of hereditary colorectal cancer (CRC), accounting for about 3% of all CRC cases in the United States [1]. Despite this, LS is often underdiagnosed or unrecognized [2] To address this issue, universal tumor screening for LS among all newly diagnosed patients with CRC is becoming more common in health care settings to increase the identification of cases of LS [3, 4]. This screening is important given patients with LS are at increased risk of developing endometrial and colorectal cancers [5]. They also have higher relative risk of developing a number of other LS-associated cancers [6, 7].

Patients with LS are recommended to undergo a range of ongoing surveillance activities, which can decrease morbidity and mortality of LS-associated cancers [4]. These surveillance recommendations have included earlier and more frequent colonoscopy, consideration for transvaginal ultrasound and endometrial sampling with the possible recommendation for total hysterectomy, and clinical neurologic examination. Upper endoscopy, annual urinalysis, and pancreatic cancer screening may also be considered based on family history [8–10]. Screening guidelines continue to be updated as new research clarifies what recommendations are beneficial, but few participants receive repeat genetic counseling to update surveillance and surgical recommendations based on a patient’s specific LS gene mutation and family history.

Given the frequency, complexity (e.g., colonoscopy preparation), and cost of surveillance activities, patients with LS may be challenged to regularly follow through with recommendations and experience gaps in surveillance care. While recent initiatives have emphasized new screening programs to improve the identification of patients with LS [11–14], less is known about patient and provider experience with tracking and managing the numerous ongoing cancer surveillance recommendations once a diagnosis of LS has occurred.

We engaged in a small exploratory study of patient and provider knowledge of, and engagement with, recommended LS surveillance activities and communication with family members in an integrated healthcare system. We chose qualitative methods as they can reveal experiences, beliefs, and challenges related to engagement in complex behavior, such as ongoing cancer screening and family communication [15–18]. We conducted in-depth interviews with patients with a molecular diagnosis of LS and their providers, primarily focusing on primary care providers (PCPs), to better understand coordination and monitoring of LS surveillance activities and to identify potential barriers to adherence to care recommendations.

## Methods

### Background and study site

We conducted interviews from March–July 2015 at Kaiser Permanente Northwest (KPNW), an integrated care-delivery system serving approximately 525,000 members. As part of care, members have direct access to an internal medical genetics department without the need for referrals and all patient care is documented through a robust electronic medical record (EMR). Genetic counselors within the genetics department follow the standard diagnosis and management guidelines for LS, as recommended by National Comprehensive Cancer Network (NCCN) and other specialty experts [8, 19]. KPNW’s institutional review board approved this study.

### Recruitment and participants

Study staff (KM) conducted chart review of the EMR for indications of a LS diagnosis and identified 43 patients and 49 providers with LS patients on their care panel. Though we will refer to the patients in this study as “patients with LS”, it is important to note that not all patients with a molecular diagnosis of LS have had a diagnosis of a LS-associated cancer. Our goal was to interview roughly 20 total participants (10 patients and 10 providers), which would allow us to qualitatively explore themes [17, 18, 20]. Patients were recruited through mailed invitations and providers by email, with a follow-up from staff (JS). We deliberately recruited both genders, a range of ages, and providers with one to several LS patients on their care panel. We focused provider recruitment mostly on PCP (internal or family medicine), rather than specialists, given they are primary point of contact and care coordination for patients at KPNW. When possible, we attempted to recruit the PCPs of interviewed patients. Interviews were conducted over the phone and audio recorded. We provided a $20 gift card to patients completing the 45-60-min interview. Given clinical obligations, provider interviews were typically 20-30 min and no incentive was offered per KPNW policy.

### Data collection and analysis

We developed open-ended interview guides [14, 17, 18] based on expertise from the study team and Advisory Board (SP, LA, GW). Interview guides explored the same topical areas and employed specific probes pertinent to patient/provider experience or role. Key areas of exploration included: familiarity with LS and related surveillance recommendations; engagement and monitoring of surveillance activities; communication with family members; and barriers/facilitators to surveillance activities. A team-member (JS) trained and experienced in interviewing conducted all interviews, which were audio-recorded and professionally transcribed verbatim.

We conducted a thematic content analysis using qualitative coding and interpretation techniques [20–22] with the aid of a qualitative analysis software program (Atlas.ti). We developed a coding dictionary based on review of a subset of transcripts and discussions with the study team. Codes represented both responses to questions from the interview guide (e.g., “familiarity with recommendations”) and those naturally derived from participant comments (e.g., “worth knowing”). A team-member (JS) trained in open-coding techniques [21], coded the interviews using Atlas.ti. Using the query function of Atlas.ti, reports were generated based on coded text. These reports were reviewed multiple times to determine initial themes, which the research team discussed and reviewed against the interview transcripts. This allowed us to further refine themes and clarify interpretations of the data. Refined themes were shared again with the research team and project Advisory Board in an ongoing process until the group reached consensus on interpretation.

## Results

### Patient and provider description (Table 1)

We completed 22 in-depth interviews with patient and provider participants. We mailed 40 invitation letters to patients and none actively declined the interview. Of those patients who called in with interest or were contacted, we completed 12 interviews with 9 females and 3 males who had a mean age of 57 and an average of 17 years of membership at KPNW. Ten patients were diagnosed with LS within the KPNW system, which at the time did not have a universal tumor screening program. Five patients sought out testing for LS due to a family member with a confirmed diagnosis and 7 reported experiencing a LS related cancer prior to their molecular diagnosis of LS. The diagnosis of LS was on average 5 years prior to the interview date, ranging between 2 and 10 years.

**Table 1:** Patient and Provider Descriptions (*N* = 22)

PATIENTS (*N* = 12)		Number
Gender	Female	9
	Male	3
Average Age in Years		57
Age Range		34-77
Average Years as KPNW^a^ Member		17
Membership Years Range		1.5-30
Diagnosed with LS^b^ in KPNW		10
Initiation/Seeking of LS Diagnosis	LS testing after CRC^c^/Ovarian Cancer	7
	Initiated LS testing due to family member	5
Average Years since LS Diagnosis		5
Years since Diagnosis Range		2-10
PROVIDERS (*N* = 10)		Number
Gender	Female	5
	Male	5
Specialty Practice	Internal Medicine (IM)	6
	Family Medicine (FM)	3
	Obstetrics/ Gynecology (Obgyn)	1
Average Years at KPNW		13
Years at KPNW Range		3-30
Average # of Patients on Care Panel		1800
# of Patients on Panel Range		1450-2000
# of LS Patients on Care Panel	Only one	7
	Two - four	3

^a^Kaiser Permanente Northwest

^b^Lynch Syndrome

^c^Colorectal Cancer

We attempted to contact 49 providers and completed 10 interviews (9 PCP and 1 specialist). Providers consisted of 5 females and 5 males and included 6 Internal Medicine (IM), 3 Family Medicine (FM), and 1 obstetrics/gynecology (Obgyn) practitioner. Four of the 9 PCPs were providers for one or more interviewed patients. Providers on average had 1800 patients on their care panel and 13 years within the integrated system. According to the EMR at the time of the interview, seven providers had only one patient with LS on their panel and three had 2-4 patients. The thematic findings presented below reflect patient and provider experiences generally and are not a direct comparison between the two groups as we were unable to interview patient/provider dyads for the whole sample.

### Familiarity with LS and engagement with screening recommendations (Table 2)

#### Patients

All patients reported having an in-depth visit with the medical genetics department following their LS diagnosis or when they transferred care to KPNW. Patients described receiving a detailed explanation of their genetic test result, LS-associated cancer risks, follow-up surveillance recommendations, encouragement, assistance to inform at-risk family members, and a packet of written information. Given this, all patients had a high degree of general knowledge about LS. Ten patients (83%) could easily describe the range, type, and frequency of surveillance recommendations; while two patients (17%) were less certain.

**Table 2:** Familiarity with LS and engagement with surveillance recommendations (patient [*N* = 12]; provider [*N* = 10])

Familiarity key findings	Exemplar quotes
Patients • All (12) received education about LS and needed surveillance activities from a genetic counselor • 83% (10) easily described the types of surveillance recommendations but were less able to always articulate screening time interval	“They (genetic counselor) gave me all the information on the risk for colon cancer and additional cancers like of the stomach and bladder.” – male “So we have the combinations blood, CBC plus kidney functions. We do the internal ultrasound and transvaginal ultrasound, and then we do colonoscopies. And the endoscopy is recommended but that one is just not on my radar screen [as to when].” – female
Providers • All had heard of LS and generally understood these patients have an increased risk for colon cancer and need more frequent colonoscopy screening • 70% (7) were much less familiar with specific surveillance recommendations, while 3 described greater detailed understanding and knowledge	“I know that it is a genetic condition making people susceptible to getting polyps that turn into cancer and that it can happen at a young age – so they need to be found and monitored.” – IM PCP “On a scale of 1-10, I would say a 2 or 3 [in knowledge] as we learned about it and most of us know of it better as HNPCC…so for screenings that are required, obviously regular GI scoping.” – FM PCP “… I don’t know the recommendations quite frankly.” – IM PCP “I would say my familiarity is fair when compared to other generalists. I know it is an autosomal dominant mutation, mismatched repair genes, if I recall correctly and it is responsible, I believe, for [an increased rate] of colorectal cancer...” IM PCP
Engagement key findings	Exemplar quotes
Patients • Most described engagement with colonoscopy (10) and endoscopy (8) every 1-2 years • About half described engagement with other recommendations like urine cytology and bloodwork (e.g. kidney/liver screening) • One female obtains transvaginal ultrasound every 1-2 years with 7 others reporting total hysterectomies	“I’ve had colonoscopy exams about every 2 years and endoscope down the throat exams at the same time.” – male “I’ve done colonoscopy every year, or sometimes I push it out to 15 months, I think that is the furthest I ever went out with it.” – female “And I also [do] bladder, abdominal ultrasound exams and another urine exam every couple of years…” – male “I scheduled the appointment with the OB/GYN right away to talk with her about the hysterectomy. I knew that is what I wanted.” – female
Providers • 70% (7) providers viewed LS as an infrequently encountered condition recalling only 1 patient on their large care panels • 3 providers with 2-4 identified patients on their care panel described regular engagement with family history documentation and follow up conversations	“I don’t have a lot of knowledge or experience with it at all… I think I just have one patient that I know of with Lynch syndrome and she told that she was diagnosed with that… I mean it is such a rare thing, you don’t come across it that often.” - IM PCP “I don’t know a lot about Lynch Syndrome. I mean it is always something I have to look up…” - IM PCP “I do know about it. I have a number of patients on my practice who have LS…I take a three-generation family history on every patient…if you have those clusters of cancers then I’m going to think maybe you have a familial cancer syndrome.” – FM PCP

*IM* internal medicine provider, *FM* family medicine provider

All patients reported adherence to colon surveillance, with ten patients (83%) obtaining a colonoscopy every 1-2 years, one (8%) every 2-3 years, and one patient (8%) who had undergone a colectomy described receiving flexible sigmoidoscopy yearly. Eight patients (67%) described obtaining upper endoscopy every 1-2 years. The remaining four participants (33%) had not yet obtained their first recommended upper endoscopy. They expressed the coordination of the upper endoscopy, along with the frequency of their colonoscopy, as complex to coordinate and felt uncertain if the upper endoscopy should be completed at the same time or in the opposite year of their colonoscopy. Additionally, for two of these four patients, both female and diagnosed with LS 6 to 10 years prior, they felt less sure of the screening frequency of upper endoscopy and generally did not have it “on the radar screen” like colonoscopy. Among the female patients, one described obtaining transvaginal ultrasound every 1-2 years and seven self-reported undergoing a total hysterectomy. About half of the patients described engagement with other recommendations such as urine cytology, abdominal CT ultrasound, and blood work for kidney and liver screening.

#### Providers

Seven providers (70%) were generally aware of LS, having heard about it more often as hereditary non-polyposis colorectal cancer (HNPCC) and learning about it briefly in medical school. While providers were much less familiar with specific LS surveillance recommendations, they understood patients were at an increased risk for CRC and often needed more frequent colonoscopy screening. These providers viewed LS as a rare condition infrequently encountered in their practice. The other 3 providers (30%) had increased familiarity with LS, detailing more specific understanding of LS and surveillance recommendations. These three providers had 2-4 patients with LS on their care panel and described how they proactively capture detailed family history, often looking for information that might indicate the need for a referral to the medical genetics department.

### Approach to and support with surveillance recommendations (Table 3)

#### Patients

While all patients described themselves as proactive about their LS care, they described important differences in the support they received from their providers. Eight patients (67%) described assistance from PCPs and specialists in tracking care needs. For instance, PCPs typically reminded them to obtain blood or lab work; and specialists reminded them about procedures such as upper and lower endoscopies. One-third (4) of the patients described taking sole responsibility for tracking their recommended care and intervals. Two of these patients did not recall ever receiving any monitoring or reminding support from their providers. While five patients reported some follow-up communication with the genetics department after their initial diagnosis in the form of a visit or telephone check-in, 7 (58%) had not. When asked why they had not experienced additional follow-up with genetics, these patients said they felt it was either unnecessary due to the thoroughness of information provided initially, that no one had told them to follow up, or that they assumed the genetics department would contact them if necessary.

**Table 3:** Monitoring of / support with LS surveillance recommendations (patient [*N* = 12]; provider [*N* = 10])

Monitoring and support key findings	Exemplar quotes
Patients • Two-thirds (8) relied on a combination of their own tracking and some type of reminder prompting from a provider • One-third (4) relied solely on their own efforts to track and complete surveillance activities • 58% (7) had not returned to genetic counselors or communicated again with specialists since their LS identification regarding surveillance recommendations	“The surgeon’s office does a really good job of reminding me when I need to do my colonoscopy - he’ll say it needs to be repeated in 6 or 12 months and I will write it on my calendar.” – male “I watch it myself for the colonoscopy. Then my PCP reminds me to go in for urine cytology check and set up for the abdominal ultrasound.” – male “I can keep track… I don’t know that I’ve gotten reminder letters about a specific test…” – female “I haven’t actually returned to the genetics department. I didn’t even think about it.” – female
Providers • 80% (8) described relying heavily on their patient and/or specialists (GI, genetic counselors) to be the experts on surveillance • 5 relied solely on specialty providers to actively monitor and follow up on their identified LS patients’ surveillance needs • 5 others described more of a partnership working with specialist departments to track, monitor, and send reminders to their identified LS participants regarding surveillance needs	“She [patient] is incredibly proactive and very educated. So she’s taking care of herself, essentially. She has a GI that she sees regularly. She really doesn’t need anything from me, because she’s so on top of it…” – IM PCP “I don’t do much… they are all followed [by] GI pretty much.” - FM PCP “…that [tracking and reminding] is done through the genetics department…” – IM PCP “I’m trying to remember to review that with them and make sure they’re doing their follow-up. And it helps to have the system [specialists] working on your side too.” - IM PCP “…between [patient], GI and myself, we kind of tack it – anything the GI indicates I document in the problem list so I can easily find it again later when she is due. So we work together as a team.” – FM PCP

*IM* internal medicine provider, *FM* family medicine provider

#### Providers

Most providers (8) offered minimal educational support or communication about LS to their affected patients, believing this would be “covered” by the medical genetics department. Two providers regularly checked in with patients during office visits about their LS diagnosis, changes in health or family history, and surveillance activities. Half of the providers described taking an active partnership role with specialists and the patient to monitor and encourage completion. The other 5 providers described relying primarily on the genetic or GI departments to track and coordinate surveillance.

### Facilitators and barriers to care coordination and receipt (Table 4)

#### Patients

All patients believed their overall LS care was coordinated and felt generally supported by their providers. They appreciated the ease of access to genetic counselors and their helpful guidance in understanding the condition, follow up recommendations, and communicating results to other family members. Additionally, almost half (5) felt their health insurance coverage facilitated ongoing surveillance as the financial burden of repeated screenings was minimal. Eight patients viewed a range of providers (PCP, GI, genetic counselor, oncologist, Obgyn) to be their core LS care team. Four felt one specialist, such as GI or oncology, to be primarily responsible.

**Table 4:** Facilitators and barriers to LS surveillance care coordination (patient [*N* = 12]; provider [*N* = 10])

Facilitators to surveillance key findings	Exemplar quotes
Patients • Overall, all felt well-supported by the health system regarding their LS related care (diagnosis, education, surveillance) • 58% (7) were particularly satisfied with the support received from the genetics department regarding communication with and education of identified patients’ family members • 42% (5) cited having comprehensive health insurance with minimal co-payments as relieving potential financial burden of the frequent surveillance activities	“I’ve been really pleased so far because everything has been so open and shared within one medical record…and I know I’m being proactive with the help of a good medical team I have in place. It is not questioned anymore about why I’m doing the tests [e.g. frequent colonoscopy] that I’m doing.” – female “People in the genetics department were very helpful helping me help my daughter find people [to screen for LS], because she is outside the system.” – female “It really makes me humble I have this wonderful insurance. I don’t know how [other] people out there pay for these procedures – that would be a challenge.” – female
Providers • All felt generally well-supported by the medical genetics department and other specialists regarding their expertise with helping LS patients • Half (5) of the providers could easily see and access LS surveillance recommendations from specialists in a preferred area of the electronic medical record, called the ‘problem list’	“I often refer to the Genetics Department – [it] is a great department…they take care of the counseling and informing on the inheritance pattern of it and who else is at risk”. – IM PCP “I think the GI Department does a really good job of population management.” – FM PCP “So it looks like [patient] recently saw GI, and had some recommendation has been updated, saying they should have a colonoscopy every one to two years so that helps... now it is in the problem list, so if somebody sees that they will know that that needs to be followed up on.” – IM PCP “I get the email reminders about those follow-up screenings needed every year. Usually [patient] gets an ultrasound, lab-work, a gynecological exam once a year, and she also gets cancer screening and upper and lower endoscopy periodically. I usually get reminders about her and do outreach calls”. – IM PCP
Barriers to surveillance key findings	Exemplar quotes
Patients • 42% (5) identified challenges in finding providers to work with that know about and understand LS and related screening criteria or being able to access the same provider (e.g. same genetic counselor) • A quarter (3) cited frequent colonoscopy preparation as burdensome • 3 also felt there was a lack of routine communication from the health system on about LS and related care	“Providers go, ‘Well now that you have Lynch you’re probably going to know a lot more about what is out there than us because you’re going to be actively researching it.’ I get that, but you are my healthcare provider, so I’d like a little bit of assistance from you too.” – female “Going through the colonoscopy is not that big of a deal but it also is – the prep for it is a lot of work and not very pleasant either!” – male “I’ve had so many of them [providers] ask me specifics on it – ‘I’ve heard about this but what exactly am I looking at?’ And then they had to go back and look – I try and give them so much information on why I’m doing [frequent colonoscopies], and some doctors question why [we] keep doing colonoscopies. I mean, they haven’t had anything found, so why do they keep requesting them…” - female
Providers • Most (9) viewed LS as a complex topic and rare topic area • Most (9) described difficulty knowing exactly who on their care panel has LS given no discrete EMR flag exists • Half (5) indicated placement of LS surveillance recommendations by specialists varies in location and is often buried in areas of the EMR that are difficult to efficiently view or search	“The fact I have one patient out of fifteen hundred [makes] it feel to me like it is not a common enough syndrome – and it is complex enough that I don’t think it is realistic to do proper Lynch Syndrome screening and surveillance in primary care.” – IM PCP “I think the communication to let us know if we have a patient with Lynch Syndrome is very important and if they’ve been referred to seeing a geneticists [yet]…what would be helpful is a clear way to know this patient has this…” – IM PCP . “The problem list is the thing that makes all of us [aware]… it is our shared medical record but it is searchable. So, if something is in the progress note, that is great, but when you are looking at a chart that has thousands of things in it, you don’t have time to search through progress notes to find the genetic note to tell you what to do.” – FM PCP

*IM* internal medicine provider, *FM* family medicine provider

One-third (4) of the patients experienced difficulty in identifying providers who were knowledgeable about LS and would not question the need for increased colonoscopy frequency. They described having to be the “expert” on LS and related recommendations, and felt uncertain who the “main” provider should be for coordinating their LS care. Three patients described the frequency of colonoscopy and related preparation to be a hindrance to timely completion of this screening. Some patients (3) felt there was a lack of routine communication from the health system about LS updates or reminders for screening.

#### Providers

All providers felt supported by the medical genetics department and appreciated their accessibility, expertise, and guidance. Half of the PCPs were not always sure who on their care panel had a LS diagnosis and could not recall their surveillance recommendations. Providers described how this information can be difficult to find, is often “buried” in specialists’ notes within the EMR, and that proactive electronic communication from specialists about LS and recommendations varies. The other 5 providers, however, indicated easily finding clear documentation in the patient chart about LS recommendations. Providers believe LS to be a complex condition that they are less familiar with and rarely encounter on their large care panels. They expressed concern that patients who do not come in or make contact via a discrete visit could potentially “fall through the cracks”. All providers described documentation of the LS diagnosis and related recommendations in a commonly utilized and viewed section of the EMR called the “problem list” as lacking consistency.

### Informing and communicating with family members

#### Patients

All patients reported sharing information about LS with living blood relatives and encouraging family members to discuss options with their providers. Patients described receiving assistance from the medical genetics department including either informing family members directly or crafting a communication letter. Patients reported informing several types of family members, including siblings, adult children, parents, cousins, nieces/nephews, and aunt/uncles.

Regarding outcomes, 92% of the patients (11) reported having at least one family member that sought LS testing. One patient reported that no one in their family sought out LS testing, but several had started colonoscopy screening based on being informed. Patients described the actions and outcomes of 54 different relatives after being informed about the possibility of a LS mutation. For these 54 different relatives, 22 were tested for LS and 11 had a positive test result; an additional 22 had yet to seek testing but two of these were preparing to test soon; and for 10 the outcomes or actions were not known to the interviewed patient.

Patients described the most common barriers for their family members in seeking LS testing included: lack of insurance coverage/inability to afford testing; difficulty either finding or negotiating the test in the given area or health system; and fear of result. Even with these barriers, three patients indicated they attempt regular follow up with family members to encourage and remind them of the importance of testing for the known familial mutation and/or altering frequency of colonoscopies.

#### Providers

Providers had much less to report about supporting patients to inform family members. Seven providers did not help communicate with or outreach to the patients’ family members primarily because the patient did not ask for this assistance or providers felt this was covered by the genetics department. Three providers recalled their patient asking them questions about informing family members. These providers discussed general inheritance issues with the patient, encouraged informing the family members, and referred their patient to medical genetics for assistance.

### Overall reactions

#### Patients

Regarding their goals and emotional state prior to the LS diagnosis, 67% (8) of the patients reflected wanting to confirm some type of hereditary risk. Four patients had initial resistance and fear about learning this genetic information and described having to be convinced to obtain LS testing. Following their LS diagnosis, most patients (10) were satisfied with receiving the “positive result”, with half finding the information a “relief” and the other half finding it initially scary and emotional, but eventually important information to know. While two participants described initial regret for obtaining the diagnosis, all of the patients expressed gratitude for knowing their LS status. Patients valued the knowledge so they could stay proactive with their own health through engagement in the surveillance activities. Patients expressed appreciation for being able to inform family members, including the opportunity to potentially reduce cancers in current or future generations.

#### Providers

All providers acknowledged the importance of identifying LS patients and engaging them in follow up surveillance activities. They felt strongly that while PCPs should be a partner in this effort, they should not be the primary point of contact or responsibility. All providers interviewed felt identification of LS and management of follow up surveillance should be coordinated by a dedicated team of specialists (e.g. genetic counselors, GI) with the PCP providing a supportive role. PCPs felt appropriate activities for themselves would entail: helping to identify/document family cancer history and placing referral to genetics department when appropriate; serving as a back up to remind patients to complete needed surveillance activities (e.g., being notified when patients are non-compliant); and encouraging LS patients to inform family members, referring to the genetics department or other resources as needed.

## Discussion

For our small study, we sought to qualitatively explore both patient and provider perspectives regarding recommended surveillance activities following a LS diagnosis within an integrated health system. We interviewed patients and providers to understand their awareness of LS and surveillance recommendations, engagement with recommendations, uncover barriers to follow through with surveillance recommendations, and reveal possible areas for health system improvements.

Similar to other studies [12, 13], we found PCPs were less familiar with LS screening recommendations than their patients. As a result, providers relied on their patients and specialists (e.g., genetic counselor or GI) to be experts on the condition. While relying on patients to serve as the “expert” for less commonly encountered genetic conditions may not be uncommon [23], it does place the burden of expertise on the patient to educate providers and potentially justify type or frequency of surveillance. This finding is echoed by our patients’ desire that their providers, particularly PCPs, have greater knowledge about LS and LS-related care recommendations.

Our interviews further underscore gaps in care coordination found in other studies [12, 13, 24] that could lead to under or over screening. Under screening for LS surveillance activities may occur when the health care system and providers lack support in clear, coordinated mechanisms for tracking and completing recommended surveillance activities. This coordination is especially complicated as patients with LS require various surveillance activities at different times and with different care providers, involving different levels of effort on the part of the patient for completion (e.g. colonoscopy prep to blood draws for lab visits). While our patients described themselves as primarily proactive in tracking their LS surveillance efforts, they were still managing multiple screening activities in a given time period for which they desired more help from their providers and the healthcare delivery system in prompting and coordinating. Some patients did report receiving assistance in the form of reminders from providers, yet there was much variability in who received the reminders, which surveillance recommendation might be prompted for, and who within the health system tracks and issues the reminders. Additionally, clear coordination among PCPs and specialists was variable, including who might be providing reminders for different surveillance activities or taking “ownership” of the monitoring to ensure patients remember and complete the screening activities.

Over screening may occur when providers are not aware of their patients current LS screening recommendations, or do not facilitate regular check-ins with genetic counselors for any screening updates. On average, our patients received their screening recommendations over 5 years prior to the interview. Given this is an evolving field, LS screening recommendations have changed over time. For these patients, this is best reflected in confusion over endoscopy screening. Two were unsure of whether this was needed and 8 were actively engaging in it every other year. While endoscopy may have been regularly recommended previously [19], current guidelines do not necessarily support this, and so patients may be unknowingly over screening in this activity. Furthermore, lack of follow up communication with the medical genetics department may be exacerbating this issue, as most of our patients reported they did not feel a need to return to genetic counseling until contacted. Most providers assumed patients were regularly checking in with the genetic counselors or being outreached to by them. Our interviews identify possible coordination challenges some health systems may experience where both patients and providers assume ‘someone’ is tracking and outreaching regarding recommended screening when this may not actually be occurring, thus leading to lack of awareness of any changes in screening recommendations and potential for over or under screening.

As more individuals are confirmed to have LS, health care systems will need to develop comprehensive programs for tracking, monitoring, encouraging adherence to surveillance recommendations, and informing at -risk family members [25, 26]. PCPs play an important and necessary role in the care and support of patients with LS [27]. Our interviews reveal that relying on patients and PCPs to ensure completion of recommended surveillance activities may create gaps in care unless improvements are made in EMR documentation, provider awareness, and coordinated support across specialty departments such as GI, Obgyn and genetics. Ultimately, the coordination and standardized follow up protocols needed to ensure patients are engaging with the complex LS surveillance recommendations might be best accomplished within a high-risk cancer department involving multiple specialists, as has been established for patients with Hereditary Breast and Ovarian Cancer syndrome due to a pathogenic variant in *BRCA1* or *BRCA2* [3, 28, 29].

The small interview sample size, while not uncommon for qualitative research, may not have obtained the full range of experiences and perspectives. We specifically focused on the experiences of PCPs because this is the primary and ongoing point person for most patient care, and we had previously interviewed a range of specialists (e.g., genetic counselors, GI, oncologists, pathologist) to understand their perspectives [30]. Given the small size of the study, we were not able to explore the relationship between a patient’s specific gene change or sense of perceived risk and their adherence to surveillance. This is an important area for future exploration. We employed several strategies to improve the trustworthiness of our qualitative interpretations [17, 18, 20], including using an experienced and trained qualitative researcher (JS); employing interview guides to assure consistency of data collection across patients/providers; and using a formal, team-based approach to analysis that included transcription, coding, and multiple reviews of summarized themes.

## Conclusion

Our interviews, derived from patients and providers at a single health system, offer important information for other health systems to consider regarding management of surveillance recommendations for the growing number of patients with LS, which could reduce morbidity and mortality related CRC or other LS-associated cancers. The promise of these reductions can only be realized, however, if providers and patients are educated on, and engaged with, recommended risk-reducing activities. A comprehensive monitoring program that involves PCPs, genetic counselors, and other specialty groups and includes provider education, electronic medical record tools, and consistent reminder prompts may be key to ensuring that patients with LS consistently obtain up-to-date recommended cancer screening and risk-reducing procedures.

## References

[1] Colorectal cancer facts & figures, 2014-2016 [http://www.cancer.org/acs/groups/content/documents/document/acspc-042280.pdf]. Accessed 2016.

[2] Bonnet D, Selves J, Toulas C, Danjoux M, Duffas JP, Portier G, Kirzin S, Ghouti L, Carrere N, Suc B (2012). Simplified identification of lynch syndrome: a prospective, multicenter study. Dig Liver Dis.

[3] Sehgal R, Sheahan K, O'Connell PR, Hanly AM, Martin ST, Winter DC (2014). Lynch syndrome: an updated review. Genes (Basel).

[4] Cohen SA, Laurino M, Bowen DJ, Upton MP, Pritchard C, Hisama F, Jarvik G, Fichera A, Sjoding B, Bennett RL (2016). Initiation of universal tumor screening for lynch syndrome in colorectal cancer patients as a model for the implementation of genetic information into clinical oncology practice. Cancer.

[5] Kohlmann W, Gruber SB, Pagon RA, Adam MP, Ardinger HH, Wallace SE, Amemiya A, LJH B, Bird TD, Fong CT, Mefford HC, RJH S, Stephens K (1993). Lynch syndrome. GeneReviews(R).

[6] Stoffel EM, Kastrinos F (2014). Familial colorectal cancer, beyond lynch syndrome. Clin Gastroenterol Hepatol.

[7] Palomaki GE, McClain MR, Melillo S, Hampel HL, Thibodeau SN (2009). EGAPP supplementary evidence review: DNA testing strategies aimed at reducing morbidity and mortality from lynch syndrome. Genet Med.

[8] Syngal S, Brand RE, Church JM, Giardiello FM, Hampel HL, Burt RW (2015). ACG clinical guideline: genetic testing and management of hereditary gastrointestinal cancer syndromes. Am J Gastroenterol.

[9] Stoffel E, Mukherjee B, Raymond VM, Tayob N, Kastrinos F, Sparr J, Wang F, Bandipalliam P, Syngal S, Gruber SB (2009). Calculation of risk of colorectal and endometrial cancer among patients with lynch syndrome. Gastroenterology.

[10] Barrow E, Robinson L, Alduaij W, Shenton A, Clancy T, Lalloo F, Hill J, Evans DG (2009). Cumulative lifetime incidence of extracolonic cancers in lynch syndrome: a report of 121 families with proven mutations. Clin Genet.

[11] Cragun D, DeBate RD, Vadaparampil ST, Baldwin J, Hampel H, Pal T (2014). Comparing universal lynch syndrome tumor-screening programs to evaluate associations between implementation strategies and patient follow-through. Genet Med.

[12] Watkins KE, Way CY, Fiander JJ, Meadus RJ, Esplen MJ, Green JS, Ludlow VC, Etchegary HA, Parfrey PS (2011). Lynch syndrome: barriers to and facilitators of screening and disease management. Hered Cancer Clin Pract.

[13] Burton-Chase AM, Hovick SR, Sun CC, Boyd-Rogers S, Lynch PM, Lu KH, Peterson SK (2014). Gynecologic cancer screening and communication with health care providers in women with lynch syndrome. Clin Genet.

[14] Cohen SA (2014). Current lynch syndrome tumor screening practices: a survey of genetic counselors. J Genet Couns.

[15] Peres J (2010). To screen or not to screen for lynch syndrome. J Natl Cancer Inst.

[16] Hall MJ (2010). Counterpoint: implementing population genetic screening for lynch syndrome among newly diagnosed colorectal cancer patients--will the ends justify the means?. J Natl Compr Cancer Netw.

[17] Denzin N, Lincoln Y (2011). The sage handbook of qualitative research.

[18] Silverman D (2009). Doing qualitative research.

[19] Provenzale D, Gupta S, Ahnen DJ, Bray T, Cannon JA, Cooper G, David DS, Early DS, Erwin D, Ford JM (2016). Genetic/familial high-risk assessment: colorectal version 1.2016, NCCN clinical practice guidelines in oncology. J Natl Compr Cancer Netw.

[20] Patton MQ (2002). Qualitative research and evaluation methods (3rd ed).

[21] Strauss A, Corbin J (2008). Basics of qualitative research: techniques and procedures for developing grounded theory.

[22] Bernard H, Ryan G (2010). Analyzing qualitative data: systematic approaches.

[23] Carroll JC, Makuwaza T, Manca DP, Sopcak N, Permaul JA, O'Brien MA, Heisey R, Eisenhauer EA, Easley J, Krzyzanowska MK (2016). Primary care providers’ experiences with and perceptions of personalized genomic medicine. Can Fam Physician.

[24] Patel SG, Ahnen DJ, Kinney AY, Horick N, Finkelstein DM, Hill DA, Lindor NM, MaCrae F, Lowery JT (2016). Knowledge and uptake of genetic counseling and Colonoscopic screening among individuals at increased risk for lynch syndrome and their Endoscopists from the family health promotion project. Am J Gastroenterol.

[25] Sperber NR, Andrews SM, Voils CI, Green GL, Provenzale D, Knight S. Barriers and Facilitators to Adoption of Genomic Services for Colorectal Care within the Veterans Health Administration. J Pers Med. 2016;6:16. 10.3390/jpm6020016 PMC4932463 27136589

[26] Hamilton AB, Oishi S, Yano EM, Gammage CE, Marshall NJ, Scheuner MT (2014). Factors influencing organizational adoption and implementation of clinical genetic services. Genet Med.

[27] Tyler CV, Snyder CW (2006). Cancer risk assessment: examining the family physician's role. J Am Board Fam Med.

[28] Cadiz F, Kuerer HM, Puga J, Camacho J, Cunill E, Arun B (2013). Establishing a program for individuals at high risk for breast cancer. J Cancer.

[29] Daly MB, Stearman B, Masny A, Sein E, Mazzoni S (2005). How to establish a high-risk cancer genetics clinic: limitations and successes. Curr Oncol Rep.

[30] Schneider JL, Davis J, Kauffman TL, Reiss JA, McGinley C, Arnold K, Zepp J, Gilmore M, Muessig KR, Syngal S (2016). Stakeholder perspectives on implementing a universal lynch syndrome screening program: a qualitative study of early barriers and facilitators. Genet Med.

